# Cardiac Magnetic Resonance in Fabry Disease: Morphological, Functional, and Tissue Features

**DOI:** 10.3390/diagnostics12112652

**Published:** 2022-11-01

**Authors:** Giovanni Donato Aquaro, Carmelo De Gori, Lorenzo Faggioni, Maria Luisa Parisella, Giacomo Aringhieri, Dania Cioni, Riccardo Lencioni, Emanuele Neri

**Affiliations:** Academic Radiology, University of Pisa, 56124 Pisa, Italy

**Keywords:** Fabry disease, cardiac magnetic resonance, T1 mapping, late gadolinium enhancement, feature tracking

## Abstract

Fabry disease (FD) is an X-linked inheritable storage disease caused by a deficiency of alpha-galactosidase causing lysosomal overload of sphingolipids. FD cardiomyopathy is characterized by left ventricular (LV) hypertrophy and should be considered in differential diagnosis with all the other causes of LV hypertrophy. An early diagnosis of FD is very important because the enzyme replacement therapy (ERT) may change the fate of patients by blocking both cardiac and systemic involvement and improving prognosis. Diagnosis may be relatively easy in young patients with the typical signs and symptoms of FD, but in male patients with late onset of disease and in females, diagnosis may be very challenging. Morphological and functional aspects are not specific to FD, which cannot be diagnosed or excluded by echocardiography. Cardiac magnetic resonance (CMR) with tissue characterization capability is an accurate technique for the differential diagnosis of LV hypertrophy. The finding of decreased myocardial T1 value in LV hypertrophy is specific to FD. Late gadolinium enhancement (LGE) is found in the late stage of the disease, but it is useful to predict the cardiac response to ERT and to stratify the prognosis.

## 1. Introduction

Anderson–Fabry disease, or simply Fabry disease (FD), is an X-linked inheritable disease caused by a deficiency of alpha-galactosidase-A enzyme. A deficiency of alpha-galactosidase causes lysosomal overload of globotriaosylceramide, which is responsible for the clinical manifestations of this disease [1]. Male patients with pathogenic mutation are all affected by disease, but the age onset of symptoms and the severity of presentation depends on the residual enzymatic activity of alpha-galactosidase. If the enzyme activity is less than 3%, the onset of symptoms is early in childhood and with more severe presentation [2]. The main manifestations involve the nervous system, the heart, kidneys, and skin. In childhood and teenage years, symptoms of the peripheral nervous system are frequent, presenting with neuropathic pain, anhidrosis (more rarely hyperhidrosis), which is associated with febrile crisis and with self-limitation of physical effort, gastrointestinal symptoms, and hearing loss [3]. Some ophthalmologic signs, such as cornea verticillate and tortuous retinal vessels, are often observed, as well as the presence of cutaneous angiokeratomas, often presenting a “swim-suit” distribution. Microalbuminuria is the first manifestation of renal involvement. For the natural history of FD, the involvement of organs becomes more evident in the second decade of life. Stroke or transient ischemic attack frequently occurs at this age. A progression from micro- to macro-albuminuria with a decrease of the glomerular filtration rate is observed, and chronic renal insufficiency is usually evident from the third decade of life [4]. Heart involvement is characterized by left ventricular (LV) hypertrophy, progressively leading to heart failure and malignant arrhythmic events.

All the signs and symptoms in childhood and teenage years should raise the suspicion of FD and induce clinicians to perform an alpha-galactosidase enzymatic activity test on plasma or the blood pool, and confirm the diagnosis with a genetic test. Enzymatic replacement therapy (ERT) is an etiological therapy that blocks the progression of disease, often with a regression of LV hypertrophy and improvement of the prognosis of the patients [5].

In some subgroups of patients, the diagnosis may be very challenging: (a) male patients with a residual enzymatic activity > 3% and <30%; (b) in females.

In male patients, when the residual enzymatic activity is >3% and <30%, FD may have a late onset with the absence of the typical signs and symptoms of childhood [1]. In this subgroup of male patients, FD may manifest in older age with complications of LV hypertrophy, stroke, or renal insufficiency. All of these manifestations may be considered typical of older ages and the diagnosis of FD never performed. This misdiagnosis could affect the patients and all of the family members carrying the mutation.

In female patients, FD is even more insidious. The organ involvement in female patients is dependent on the lyonization phenomenon: in each cell of females one X-chromosome is inactivated and compacted in the Barr body. Because of this phenomenon, female patients may be completely asymptomatic or present with severe organ involvement in the case of inactivation of the normal chromosome [6]. Heart involvement could be the only manifestation of this disease in females.

The first suspicion of FD in male patients with late onset presentation and in females with lone cardiac involvement is usually provided by cardiac magnetic resonance (CMR).

CMR is the preferred imaging technique to evaluate the phenotype of cardiomyopathies. CMR provides the differential diagnosis among the causes of LV hypertrophy through an accurate assessment of morphological, functional, and tissue characteristics. In a recent study [7], CMR reclassified the initial echocardiographic etiologic suspicion in 43% of patients with LV hypertrophy. This reclassification of a diagnosis has important implications for the clinical management of patients and for prognostic evaluation. CMR has the unique capability to detect sphingolipids overload by using the T1 mapping technique and to provide prognostic information through the assessment of myocardial fibrosis with the late gadolinium enhancement (LGE) technique [8]. The aim of this review is to discuss the role of CMR in FD.

## 2. Morphological Features of Fabry Disease

As in other organs, cardiac involvement of FD is characterized by lysosomal overload of sphingolipids in myocytes. In the left ventricle, the effect of such sphingolipids overload causes wall thickening and hypertrophy [9]. Sphingolipids deposit is homogeneous in LV myocardium, and hypertrophy is usually described as concentric [10]. However, asymmetric LV hypertrophy involving the interventricular septum or the LV apex was also described in FD.

CMR is more accurate than echocardiography for the evaluation of regional wall thickness and to define the pattern of hypertrophy [11,12]. CMR does not suffer from the acoustic window limitations, it is a multiplanar technique allowing the measurement of wall thickness in planes orthogonal to myocardium and with a better depiction of both the endocardial and epicardial border. In Table 1 are shown the CMR and echocardiographic studies reporting the pattern of LV hypertrophy in FD [9,10,13,14,15,16,17,18,19,20,21]. Globally, 736 patients with FD were included in those studies, demonstrating a prevalence of LV hypertrophy in 58% of cases (426 patients). The majority of patients (n = 388 patients, 91%) had concentric hypertrophy, whereas an asymmetrical septal hypertrophy was found in 8% (n = 35) and an apical pattern in 3 (1%). The practical information obtained by these data is that the finding of asymmetrical LV hypertrophy, that is generally considered a hallmark of the sarcomeric hypertrophic cardiomyopathy, cannot be used as a diagnostic criterion to exclude Fabry disease. Furthermore, considering the great prevalence of sarcomeric hypertrophic cardiomyopathy in the general population, the coexistence of both Fabry and sarcomeric diseases is also possible [11].

The development of LV hypertrophy is associated with the severity of the presentation of Fabry disease. It may occur later in a patient’s life, particularly in females. In the study by Nieman et al., evaluating a cohort of FD patients with an average age of 42 years, LV hypertrophy was found in 17% of females and in 65% of males [6].

Another morphological feature often associated with FD, is the hypertrophy of papillary muscles. The involvement of papillary muscles is more evident in FD than in other causes of LV hypertrophy [22,23,24,25]. The evaluation of papillary muscle hypertrophy may be difficult, and different methods of measurement were proposed: maximal papillary muscle diameter, papillary muscle mass or transverse area, and the ratio between papillary muscle area and the LV transversal circumference. However, papillary muscles hypertrophy is not specific of FD, and was reported in 14% of patients with other conditions [24].

## 3. Functional Features of Fabry Disease

FD is not usually associated with LV systolic dysfunction. A more complex evaluation of regional and global systolic function is performed by feature tracking analysis. Using this technique, data of myocardial global longitudinal strain (GLS), global circumferential strain (GCS), and global radial strain (GRS) are obtained from conventional cine images. Only a few studies have evaluated myocardial strain with CMR in FD, even with discordant results. In the study by Mathur et al., no significant differences of GLS were found between patients with FD and healthy controls [26]. In contrast, in the study by Augusto et al., FD patients had worse GLS than controls, even considering the group of FD patients without LV hypertrophy and with normal T1 values [24]. A loss of the base-to-apex CS and LS gradient was also described in the early stage of FD [27]. However, this finding is not specific to FD, being found in other cardiomyopathies, such as in cardiac amyloidosis [28].

Diastolic LV dysfunction is also possible in FD, caused by the increased stiffness of hypertrophic myocardium. In the advanced stage, left atrial dilation and atrial fibrillation may occur. For this, FD is not different from other causes of LV hypertrophy.

## 4. T1 and T2 Mapping in Fabry Disease

FD is probably the most important and robust indication for T1 mapping. Native T1 mapping consists of the measurement of myocardial T1 in basal conditions. The T1 of normal myocardium should be constant at the same magnetic field intensity and any change of T1 is related to disease conditions [29]. T1 is the longitudinal relaxation time, that is, the time constant expressing the exponential recovery of longitudinal magnetization, passing from a high energy, non-equilibrium state, induced by the radiofrequency pulse, to a state of thermodynamic equilibrium. The shorter the T1, the faster the recovery of longitudinal magnetization. T1 differences are based on the difference among the molecules containing hydrogen protons (spin-lattice): hydrogen protons in long-chain triglycerides of fat tissue have a very short T1, whereas the free-water hydrogen has the longest values. Myocardial T1 has intermediate values ranging from 880 to 1150 ms at 1.5 T, depending on the sarcomeric protein concentration, glycogen content, and water content [30].

**Figure 1:**
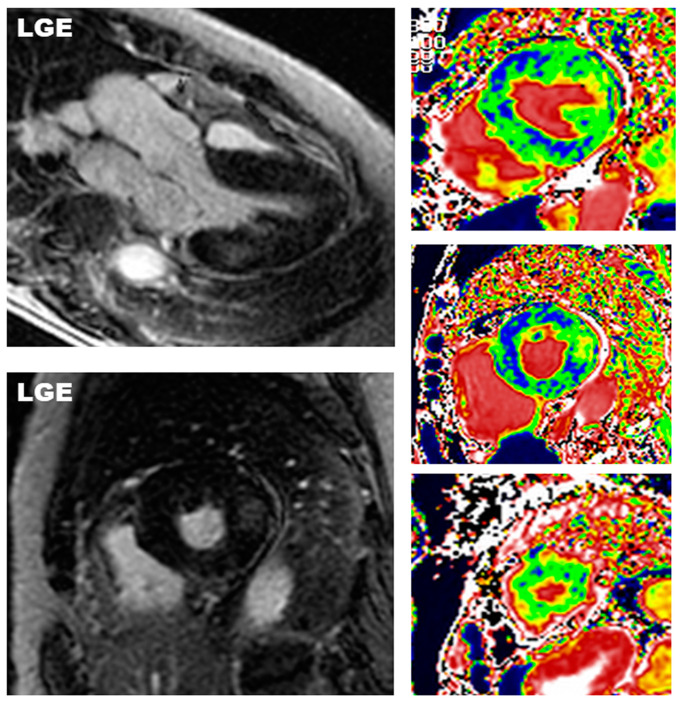
CMR of Fabry disease. Images of a 65-year-old female with Fabry disease and concentric left ventricular hypertrophy and typical CMR presentation. On post contrast image (four-chamber and short axis views in left panels), area of late gadolinium enhancement (LGE) is found in the mid-wall layer of the inferolateral wall, which is the typical site of LGE in Fabry disease. At T1 mapping (short axis views in right panels) a diffuse decrease of native myocardial T1 (blue areas) is found.

Myocardial T1 is increased for oedema, for fibrosis/scars (collagen matrix of replacement scars increases the interstitial space and water content), and for amyloidotic protein deposit. In contrast, T1 is decreased in the presence of iron overload (haemorrhagic infarction, hemochromatosis) or for fat infiltration/metaplasia (arrhythmogenic cardiomyopathy, metaplasia of old scars) [31]. The lysosomal sphingolipids deposit of FD has low T1 values, similarly to fat [32]. Among the cause of LV hypertrophy, FD is the unique condition associated with a short myocardial T1 (Figure 1). In sarcomeric hypertrophic cardiomyopathy, T1 is only patchily increased, usually in a region with coexisting fibrosis [33]. In cardiac amyloidosis, myocardial T1 is diffusely and severely increased [34]. FD may also be easily distinguished by hemochromatosis (usually not associated with LV hypertrophy), because iron overload causes a significant decrease of myocardial T2*.

In the study by Pica et al., low myocardial T1 was found in the majority of FD patients with LV hypertrophy and also in 50% of those without hypertrophy [35]. As a result, CMR may allow for an early diagnosis of FD in cases of LV hypertrophy when clinical signs and symptoms are consistent. However, caution should be exercised in patients with slightly reduced T1 values who do not have hypertrophy and neither clinical sign or symptoms of FD. Many factors, in fact, may influence the reliability of T1 measurement.

T1 mapping pulse sequences are divided into two different methods: saturation recovery (SASHA) or inversion recovery (MOLLI, ShMOLLI). Differences in pulse sequence, in the setting of pulse sequence parameters, and in the algorithm used for the fitting of the SI/time analysis curve, may result in different T1 values. A patient’s characteristics, such as heart rate, ability to hold breath, and body dimensions, may also influence the measurement [29]. Finally, different MRI machines (even by the same vendors and of the same model and release) may produce different T1 measurement because of shimming and magnetic field inhomogeneity that cannot be modeled and corrected. For these reasons, the Society for Cardiovascular Magnetic Resonance (SCMR) position paper indicates that every institution should have its on-site range of normality for myocardial T1 (as well as for T2 mapping) [36]. The meta-analysis by Ponsiglione et al. analyzed all of the studies with T1 mapping analysis in FD [37] and evaluated fourteen eligible studies, including a total of 477 FD patients and 505 controls. In all of the studies, myocardial T1 was significant lower in FD than in controls. However, considering only the studies with 1.5 T, the average values of myocardial T1 ranged from 863 ± 23 ms to 1070 ± 50 ms in FD and from 938 ± 21 to 1170 ± 27 in controls with a great overlap of value between FD and controls.

The T1 mapping technique may suffer from both false positive and negative results in the diagnosis of FD. Physiological hypertrophy of athletes is associated with the increase of sarcomeric proteins and decrease of intracellular and interstitial water content. This may result in a slight reduction in myocardial T1, that, as a result of concentric hypertrophy, may mimic FD [38]. In that case, the clinical history helps for differential diagnosis.

**Figure 2:**
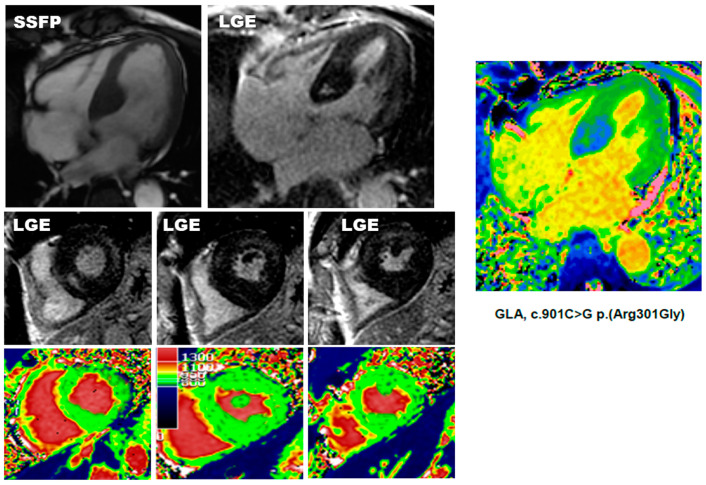
Example of pseudo-normalization of T1 in Fabry disease. CMR images of a 70-year-old male with late onset Fabry disease. The patten of LV hypertrophy is asymmetric, with wall thickening apparently limited to the basal septum. The set of three short axis T1 mapping showed myocardial T1 values within the normality range. On post-contrast images, a diffuse LGE is found in the lateral wall, apex, and even in the anteroseptal wall. Fortunately, T1 mapping was also acquired in four-chamber views and a small area of decreased T1 values was found in the basal septum, corresponding to the region of hypertrophy and with only a small island of LGE. This finding raised the suspicion of Fabry disease that was eventually confirmed by enzymatic test and genetic evaluation.

As discussed in detail in the next paragraph, the occurrence of myocardial fibrosis may alter the measurement of T1 in FD [21]. Myocardial fibrosis increases T1 and pseudo-normalizes the measurement. This is particularly evident in females, where fibrosis is often seen even before the development of LV hypertrophy [6]. The finding of “normal” T1 values despite extensive fibrosis should raise the suspicion of FD. Areas of low T1 may be distributed within myocardium, particularly in females, in the early stage of disease or in the late stage with coexisting fibrosis. Current SCMR position papers suggest the acquisition of only three short axis views of T1 mapping. With such an approach, areas of abnormal T1 could be missed [36]. The example of Figure 2, in a patient with FD and extensive fibrosis, shows areas of decreased T1 only in the basal septum of four-chamber views, which was missed in short axis images. We strongly suggest to cover all of the LV with a complete dataset of short axis images of T1 mapping from mitral annulus to LV apex or by acquisition of short axis and long axis images.

T2 mapping may have a role in the evaluation of FD. Few studies reported a diffuse increase of myocardial T2 in FD, whereas other studies found abnormal T2 only the inferolateral wall, associated with LGE [39,40]. This increase of myocardial T2 was explained as an inflammatory phenomenon accompanying myocardial damage. T2 increase is not specific to FD. In sarcomeric hypertrophic cardiomyopathy, a focal increase of myocardial T2, as well as areas of hyperintensity in conventional T2-weighted FSE, were reported [41]. Moreover, myocardial edema is seen in the acute phase of myocardial damage from many different conditions, such as ischemic heart disease, cocaine-induced myocardial damage, sarcoidosis, etc.

Both T1 and T2 mapping are useful to evaluate myocardial response to ERT. Imbriaco et al. demonstrated that ERT can normalize myocardial T2 in the region with abnormal values at basal evaluation [42]. Nordin et al., demonstrated a significant increase of myocardial T1 in patients naïve to therapy and in those with initiated ERT, whereas a substantial stability of values was found in those with long-time established therapy [43].

## 5. Late Gadolinium Enhancement and Extracellular Volume Mapping in Fabry Disease

Late gadolinium enhancement (LGE) is the most important CMR feature. Gadolinium-based media are interstitial agents, and they are not specific to fibrosis. However, the collagen matrix of scars, replacing dead myocyte, increases interstitial space and the myocardial distribution volume of gadolinium. For this reason, LGE is generally considered a valid marker of myocardial fibrosis. A pattern of presentation of LGE is very useful for the differential diagnosis among the different causes of LV hypertrophy, as well as for differential diagnosis between ischemic and non-ischemic heart disease [44]. In cardiac amyloidosis the pattern of LGE is very specific, permitting a definite diagnosis in most cases [45]. Although less specific, the LGE pattern also helps in the diagnosis of sarcomeric hypertrophic cardiomyopathy, where LGE has an important prognostic role, being a strong predictor of malignant ventricular arrhythmias [46,47].

In FD, as explained above, the intracellular overload of sphingolipids, increases myocyte dimensions and decreases interstitial space. LGE is only seen in the late stage of FD when a sufficient mass of necrotic myocytes causes macroscopic fibrosis [6]. In FD, LGE is usually located in the inferolateral basal wall of LV, with a mid-wall distribution [10]. However, this pattern of LGE is not specific to Fabry disease, being found in other conditions, such as myocarditis, arrhythmogenic cardiomyopathy, sarcoidosis, etc. [48]. In Table 2 are shown data of LGE presentation reported in FD studies [6,10,24,27,49,50,51,52,53,54,55]. The overall prevalence of LGE in FD was 33% (in 145 out of 442 patients). Eight studies also reported the distribution of LGE in LV myocardium. LGE was non-ischemic in all cases, 113 patients (94%) presented inferolateral basal mid-wall LGE, and 7 (6%) a different pattern. All of the patients of this latter group had asymmetrical LV hypertrophy, and LGE was located in hypertrophic segments (mostly in interventricular septum). Multiple myocardial areas of LGE were reported in 29 patients (24%). Interestingly, Nieman et al. demonstrated that LGE may be detected earlier than LV hypertrophy in female patients [6].

LGE is useful for the identification of the “pseudo-normalization of myocardial T1” in FD [21]. Briefly, as mentioned above, in FD, native myocardial T1 is decreased because of intracellular sphingolipids deposition. In contrast, myocardial fibrosis increases myocardial T1, compensating for the decrease of T1 by sphingolipid deposition. Then, myocardial regions containing both islands of viable myocytes (loaded with sphingolipids) and areas of fibrosis may have average T1 values within the range of normality (example in Figure 2). The identification of LGE in regions with apparently “normal” T1 values should raise the suspicion of “pseudo-normalization” of T1. Myocardial fibrosis usually does not involve all of the myocardial segments, and it is very important to measure myocardial T1 in regions spared from LGE.

LGE has an important prognostic role in FD. In patients treated with the ERT, the studies by Weidemann et al. [56] and Beer et al. [50] demonstrated that the presence of LGE was associated with a low likelihood of regression of LV hypertrophy, and with a scarce improvement of exercise capacity. Moreover, the presence of LGE was associated with a greater risk for malignant arrhythmic events, including sudden cardiac death [57].

Extracellular volume mapping (ECV) consists of the measurement of the distribution volume of gadolinium in myocardial tissue. Since gadolinium is an interstitial contrast agent, its distribution volume corresponds to the ECV of myocardium (interstitial space plus blood vessel). ECV is calculated as the ratio between myocardial and blood pool variation of R1 (that is, 1/T1) induced by gadolinium injection, corrected by the haematocrit [34]. ECV is increased in many conditions, such as amyloidosis, myocardial infarction, and generally, fibrotic myocardium of any causes. In Fabry disease, interstitial spaces are reduced by the intracellular overload of sphingolipids. Then, in the absence of fibrosis, ECV is normal or even decreased, and its role is limited to more advanced stages of disease.

## 6. CMR Protocol

The proposed CMR protocols of pulse sequence for FD used in our institution is shown in Table 3 (modified from the Society for Cardiovascular Magnetic Protocol, standardized protocols, ref. [58]).

## 7. Conclusions

Despite FD cardiomyopathy being a rare cardiac disease, it should be considered in the differential diagnosis with all of the conditions causing LV hypertrophy. An early diagnosis of FD is very important because the instauration of ERT may change the fate of the patients, reversing or blocking the development of cardiac involvement as well as of the other systemic manifestations. Moreover, the detection of a FD proband allows for the screening of other family members.

The diagnosis may be straightforward in males when specific signs and symptoms are present from childhood. However, in male patients with late onset disease and in females, the diagnosis is more challenging. Morphological and functional features do not permit for the exclusion of the diagnosis. Decreased myocardial T1 associated with LV hypertrophy is an important feature in suspecting FD. The assay of alpha-galactosidase enzymatic activity and the genetic evaluation confirm the diagnosis. Finally, LGE is important in FD to predict cardiac response to ERT and for prognostic evaluation.

## Figures and Tables

**Table 1:** Prevalence and pattern of left ventricular hypertrophy in Fabry disease.

First Author, Journal, Year of Publication (Ref.)	Patients with FD	LV Hypertrophy	Concentric Hypertrophy	Septal/Apical Hypertrophy
	**n.**	**n. (%)**	**n. (%)**	**n. (%)**
**Bass JL, *Am Heart J*, 1980 [13]**	32	15 (48%)	13 (87%)	2 (13%)/0
**Nakao N, *Eng J Med*, 1995 [14]**	7	7 (100%)	7 (100)	0 (0%)/0
**Linhart A, *Am Heart J*, 2000 [15]**	30	14 (47%)	11 (79%)	3 (21%)/0
**Sachdev B, *Circulation*, 2002 [16]**	6	6 (100%)	5 (83%)	1 (17%)/0
**Chimenti C, *Circulation*, 2004 [17]**	4	4 (100%)	2 (50%)	1 (25%)/1 (25%)
**Kawano M, *Am J Cardiol*, 2007 [18]**	13	13 (100%)	4 (31%)	9 (69%)/0
**Wu JC, *European Heart Journal*, 2010 [19]**	139	118 (85%)	115 (97%)	3 (3%)/0
**Elliott P, *Heart*, 2011 [9]**	7	7 (100%)	4 (57%)	3 (43%)/0
**Deva DP, *JCMR*, 2016 [10]**	39	22 (56%)	17 (77%)	3 (14%)/2 (5%)
**Arends M, *PLoS ONE*, 2017 [20]**	293	123 (42%)	123 (42%)	0
**Kampmann, C, *Int J Cardiol*, 2008 [21]**	166	97 (58%)	87 (52%)	10 (6%)/0
**Total**	736	426 (58%)	388 (91%)	35 (8%)/3 (1%)

**Table 2:** Prevalence and pattern of late gadolinium enhancement in Fabry disease.

First Author, Journal, Year of Publication (Ref.)	Patients with FD	Late Gadolinium Enhancement (LGE)	Classic Pattern (Basal Inferolateral Non-Ischemic LGE)	Other Patterns	Multiple Site of LGE
	**n.**	**n. (%)**	**n. (%)**	**n.**	
**Moon, *Eur. Heart J.*, 2003 [49]**	26	13 (50%)	12 (93%)	1 inferior	-
**Beer, *Am. J. Cardiol.*, 2006 [50]**	35	8 (31%)	6 (75%)	1 anterior/1 septal	-
**Pieroni, *J. Am. Coll. Cardiol.*, 2006 [51]**	40	10 (25%)	10 (100%)	-	1
**De Cobelli, *Am. J. Roentgenol.*, 2009 [52]**	13	10 (77%)	10 (100%)	-	2
**Niemann, *JACC: Cardiovasc Imaging*, 2011 [6]**	104	41 (39%)	41 (100%)	-	13
**Kozor, *Heart*, 2016 [53]**	44	15 (34.1%)	14 (93%)	1 anteroseptal	13
**Deva, *JCMR*, 2016 [10]**	39	17 (44%)	14 (76%)	2 apical/1 inferior	-
**Nojiri *J. Cardiol.*, 2020 [54]**	26	6 (23%)	6 (100%)	N/A	-
**Roller, *J. Clin. Med.*, 2021 [27]**	28	8 (28%)	N/A	N/A	N/A
**Zhao, *Cardiovasc Diagn. Ther.*, 2021 [55]**	20	4 (20%)	N/A	N/A	N/A
**Augusto, *Eur. Heart J. Cardiovasc Imaging*, 2021 [24]**	67	13 (19%)	N/A	N/A	N/A
**Total**	442	145 (33%)	113 (94%)	7 (6%)	29 (24%)

**Table 3:** CMR protocol in Fabry disease.

Techniques	Recommended Acquisition Planes	Pulse Sequence	Scope
Three-plane localizer	Axial, coronal, sagittal	Vendor specific	Heart localization, image planning
Cine-SSFP	Two-, four-, three-chamber views or radial acquisition	SSFP (FIESTA, True-FISP, Balance).	LV morphology, regional wall motion assessment
Cine-SSFP	Short axis views from mitral valve plane to LV apex	SSFP (FIESTA, True-FISP, Balance).	LV morphology, regional wall motion, quantification of ventricular volumes, mass and functional parameters
T1 mapping	Three-short axis views + three long views (two-, four-, three-chamber views) or full coverage of LV by short axis views	MOLLI, ShMOLLI, SASHA, SMART-T1	Measurement of native myocardial T1
T2 mapping	Three-short axis views	GRASE, T2-Prep SSFP, MESE	Measurement of native myocardial T2/edema detection
Gadolinium-based contrast media injection			
TI-scout	Four-chamber view	TI-scout, Cine-IR, Lock-Locker	Choice for appropriate TI for LGE
Late gadolinium enhancement	Two-, four-, three-chamber views (or radial acquisition) or full coverage of LV by short axis views	2D-LGE, 3D-LGE, PSIR	Detection of fibrosis, search for specific pattern for differential diagnosis

## Data Availability

Data is available on request to the author.
